# Supplementary material for: Designing self-tracking experiences: A qualitative study of the perceptions of barriers and facilitators to adopting digital health technology for automatic urine analysis at home
Source: PLOS Digit Health. 2023 Sep 15;2(9):e0000319. doi: 10.1371/journal.pdig.0000319 (PMC10503698; doi:10.1371/journal.pdig.0000319)
Supplement: S1 Appendix — (DOCX) [file pdig.0000319.s001.docx]

S1 Appendix: Semi-structured interview script

**Intro**

- About myself and the project
- Information sheet and consent form
- Is it ok if I record the interview
- Feel free to elaborate on any details that come to mind
- How much time do you have?

**Relationship with personal wellbeing/health**

- How old are you?
- How would you describe your attention to your personal health?
- Are you paying attention to something or taking specific actions to prevent future problems?

Why/why not?

**Use of quantified self**

- What do you use already? (voluntarily)
  - If several, do you ever wish they were integrated?
- What are you looking to find out?
- Do you look back in time at past data?
- Positive aspects
- Negative aspects / Fears
  - Have you ever found out negative information?
- Have you ever considered buying a specific tool? (ex: fitbit)
- Have you ever discovered some data you didn’t think you had tracked (ex: steps per day on your phone)?
- Do you ever get the same data multiple days in a row?
- Do you ever measure anything related to your internal health (not exercise)?
- If you had a magic quant self device, what would it measure?
- Have you got an app on your phone, would you mind showing me?

**Quantified self in relation to other people**

- Did you ever think about tracking someone else?
- Would you ever consider measuring the health of your child?
- Do you ever share the information with other people?

**Device**

- Urine analysis can tell you many things about your well being. Let’s say you have a device to check what is in your urine, how would you imagine it?
- Two scenarios
  - Something that was in your toilet? Or something more like a pregnancy test?
- Who else uses your bathroom?
  - Visitors
  - Cleaning
  - Aesthetic

**Finish**

- Thank you for your time!
- Can you explain a bit about your role at La Source?
- How much experience do you have with urine analysis?
- Hospital context
- Care home context - experience with older people
- Home context
