# Supplementary figures and images for: Designing self-tracking experiences: A qualitative study of the perceptions of barriers and facilitators to adopting digital health technology for automatic urine analysis at home
Source: PLOS Digit Health. 2023 Sep 15;2(9):e0000319. doi: 10.1371/journal.pdig.0000319 (PMC10503698; doi:10.1371/journal.pdig.0000319)

S2 Appendix: User-test questionnaire

**QUESTIONNAIRE PROTOTYPE 1 AND 2**


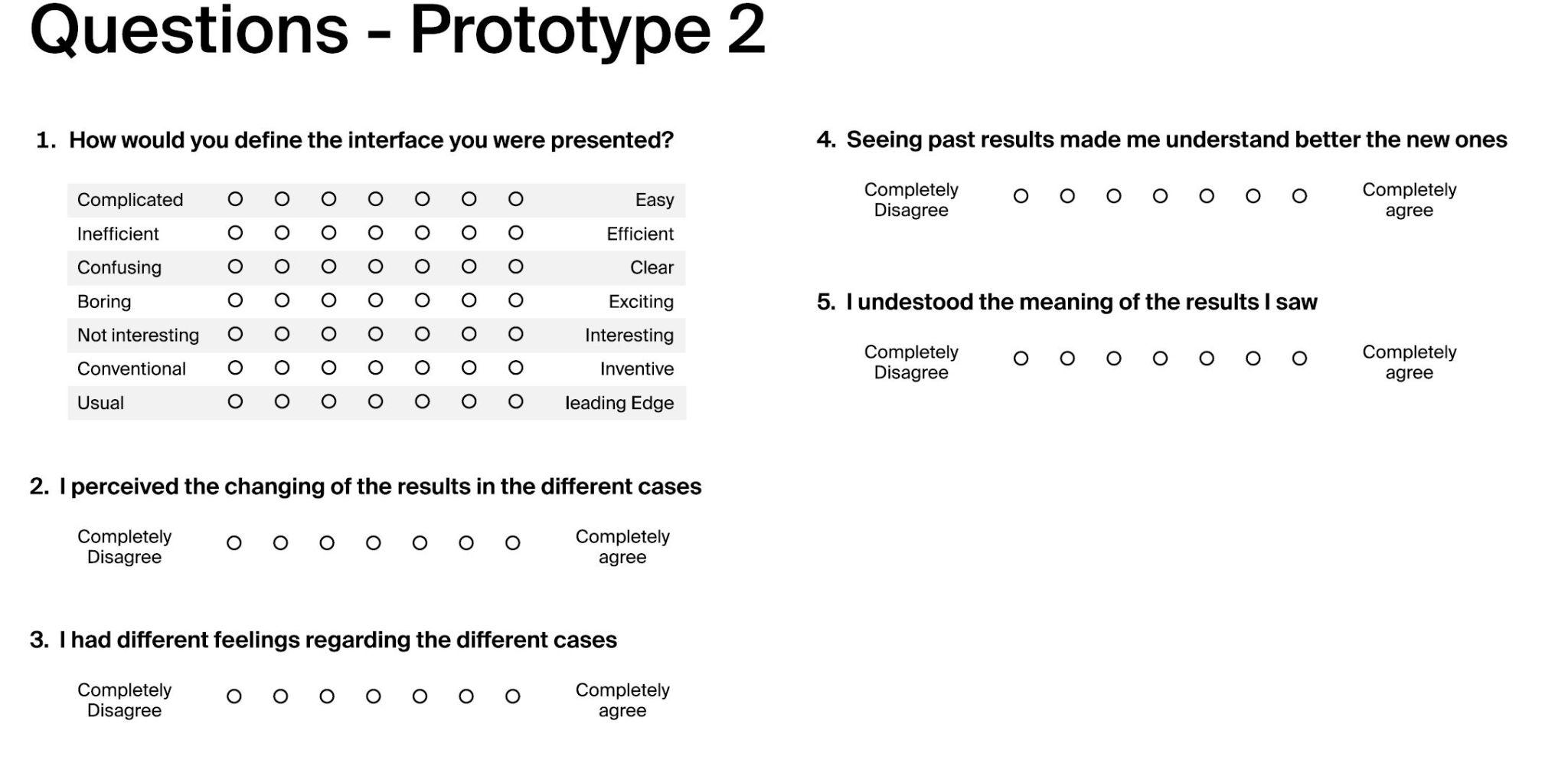


**QUESTIONNAIRE PROTOTYPE 3**


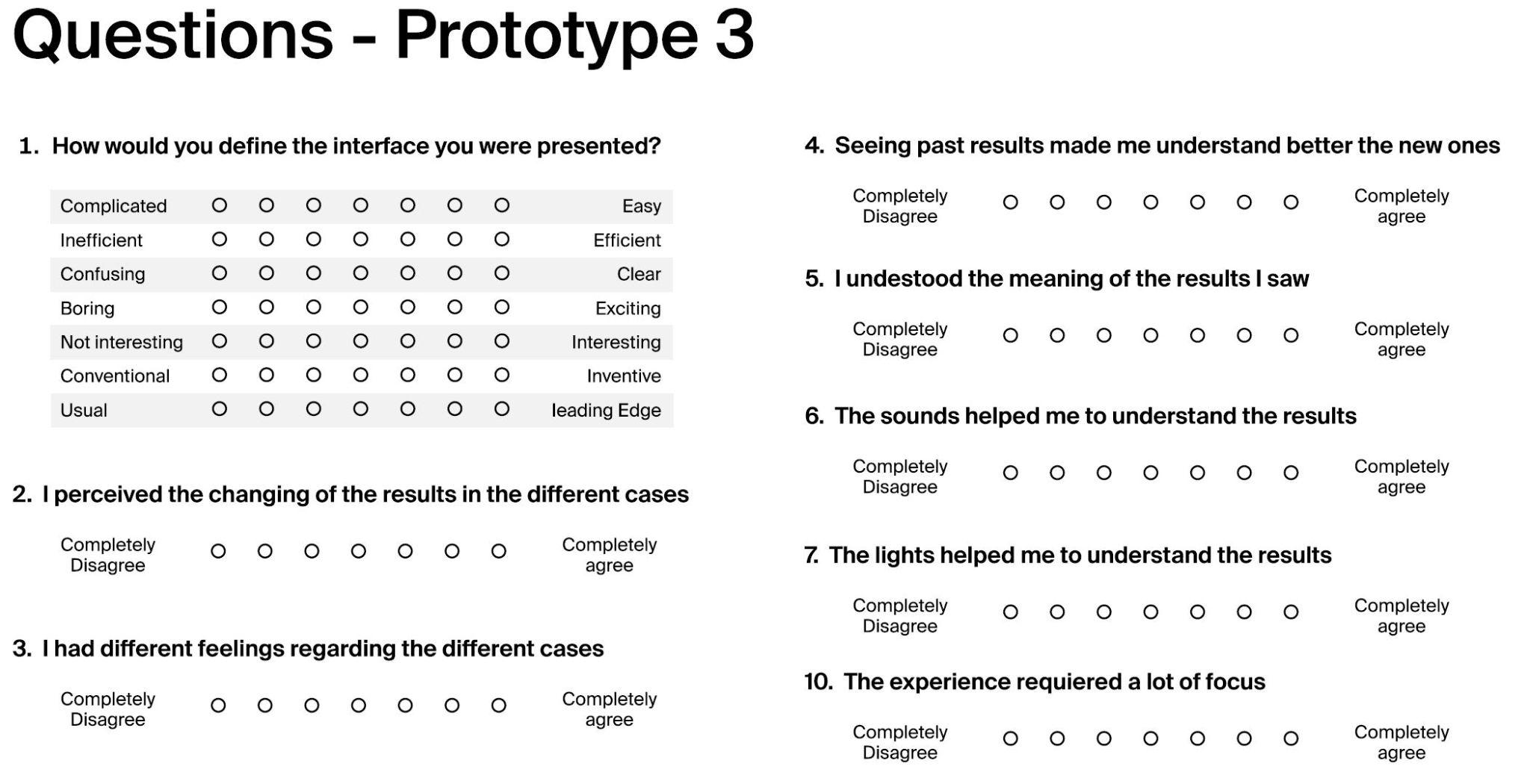

Supplement: S2 Appendix — (DOCX) [file pdig.0000319.s002.docx]
