# Supplementary material for: Designing self-tracking experiences: A qualitative study of the perceptions of barriers and facilitators to adopting digital health technology for automatic urine analysis at home
Source: PLOS Digit Health. 2023 Sep 15;2(9):e0000319. doi: 10.1371/journal.pdig.0000319 (PMC10503698; doi:10.1371/journal.pdig.0000319)
Supplement: S3 Appendix — (DOCX) [file pdig.0000319.s003.docx]

S3 Appendix: Semi-structured Interviews coding

| Theme | Details | Example | N participants |
| --- | --- | --- | --- |
| Need for guidance | Expression of desire or appreciation for recommendations relative to their data | “Here’s my result, should I do something about it?”  “A device should do more than just log” | 10/16 |
| Motivation | Idea that motivation to perform better is the main reason behind the usage of self-tracking technologies | “It really motivates me to try and go further each time” | 8/16 |
| Data sharing aversion | Expression of dislike or disinterest towards sharing their own personal data on social medial or real life | “I never even tell people I track what I eat, let alone share it. I don’t think anybody would care” | 5/16 |
| Privacy and toilet sharing | Mentions of issues of privacy or other’s people sharing of the toilet | “They already know everything, I don’t want to give something more”  “I don’t want my guests to see it and freak out!” | 6/16 |
| Effort and manipulation | Mentions of the personal effort that would be needed to use a tracking device, or the act of manipulating it and the toilet | “I used a calorie tracking app for two weeks, but it was too time consuming, complicated, and lacked joy” “I would like some level of automation, unless it’s just like a clearblue” | 5/16 |
| Usefulness | Comments regarding the urine tracking device being useful or interesting | “It could really change people’s medical routine, especially if you have something like diabetes” | 5/16 |
| Loss of interest | Mentions of participants stopping using self-tracking technologies for loss of interest | “I use them a lot in the beginning, but I quickly lose curiosity” | 3/16 |
| Data sharing liking | Expression of appreciation towards sharing their own personal data on social media or real life | “Look at me, I’m good!” | 1/16 |
| Tracking of other people aversion | Doubts or dislike regarding using the urine tracking device for tracking of others (e.g. children, parents) | “I would never use it like that I think, it feels like crossing boundaries” | 8/16 |
| Data anxiety | Idea that having access to too much personal data can bring anxiety | “Sometimes having too much information makes patients anxious” | 2/16 |
| Dealing with urine | Mentions of not being bothered or disgusted about the idea of dealing with urine in the context of-tracking | “You have to clean the toilet anyways, so what does it change?” | 8/16 |

#### 
