# Supplementary material for: Designing self-tracking experiences: A qualitative study of the perceptions of barriers and facilitators to adopting digital health technology for automatic urine analysis at home
Source: PLOS Digit Health. 2023 Sep 15;2(9):e0000319. doi: 10.1371/journal.pdig.0000319 (PMC10503698; doi:10.1371/journal.pdig.0000319)
Supplement: Attachment — Submitted filename: Response to Reviewers.docx [file pdig.0000319.s004.docx]

To whom it may concern,

Before answering to the comments brought by the editors and reviewers, we would like to express our sincere appreciation for the time and effort the internal and external experts invested in reviewing our proposal. We have carefully considered all comments and remarks and have taken them into account in the revision process. In this regard, we are convinced that the paper considerably benefited from your thoughtful suggestions, thank you very much!

## For clarity, in our response we reported the editors and reviewers’ comments in italics and our answers in bold.

*1. Please review your reference list to ensure that it is complete and correct. If you have cited papers that have been retracted, please include the rationale for doing so in the manuscript text, or remove these references and replace them with relevant current references. Any changes to the reference list should be mentioned in the rebuttal letter that accompanies your revised manuscript. If you need to cite a retracted article, indicate the article's retracted status in the References list and also include a citation and full reference for the retraction notice.*

**We have reviewed and updated our reference list so that it is complete and correct. We acknowledge that one paper precedently referenced by us has been retracted. For this reason, the paper by Wiley-Rapoport C titled “The quantified self and the psychological self: Using narrative identity to bring meaning to fitness band data” has been removed from our reference list.**

*2. Please amend your detailed Financial Disclosure statement. This is published with the article. It must therefore be completed in full sentences and contain the exact wording you wish to be published.*

*a. State the initials, alongside each funding source, of each author to receive each grant.*

*b. State what role the funders took in the study. If the funders had no role in your study, please state: “The funders had no role in study design, data collection and analysis, decision to publish, or preparation of the manuscript.”*

*c. If any authors received a salary from any of your funders, please state which authors and which funders.*

*If you did not receive any funding for this study, please simply state: “The authors received no specific funding for this work.”*

**We** **added the “Financial Disclosure statement” at the end of the manuscript.**

**We also decided to add a “Data Availability statement”, also at the end of the manuscript.**

*3. Please send a completed 'Competing Interests' statement, including any COIs declared by your co-authors. If you have no competing interests to declare, please state "The authors have declared that no competing interests exist". Otherwise please declare all competing interests beginning with the statement "I have read the journal's policy and the authors of this manuscript have the following competing interests:"*

**We** **added the “Competing Interests statement” at the end of the manuscript.**

*4. Please provide separate figure files in .tif or .eps format only and remove any figures embedded in your manuscript file. Please also ensure that all files are under our size limit of 10MB.*

**We’ve removed the figures and we provide them separately. We also added all the captions at the end of the manuscript, after the references.**

*5. Figure 3 and 5: Please confirm whether you drew the images / clip-art within the figure panels by hand. If you did not draw the images, please provide (a) a link to the source of the images or icons and their license / terms of use; or (b) written permission from the copyright holder to publish the images or icons under our CC-BY 4.0 license. Alternatively, you may replace the images with open source alternatives. See these open source resources you may use to replace images / clip-art*

**We confirm that we drew these images ourselves and no permission is needed to use them.**

6. Figure 5 includes an image of an identifiable person. Please provide written confirmation or release forms, signed by the subject(s) (or their parent/legally authorized guardian), giving permission to be photographed and to have their images published under our CC-BY 4.0 license.

Otherwise, we kindly request that you remove the photograph.

**The identifiable person in the picture is first author (Margherita Motta), who agrees with the publication of the picture.**

## Reviewer #1

*This is an excellent paper that discusses timely and significant issues related to self-tracking technologies with new empirical insights that are grounded on the emerging sociological literature on these issues.*

*I think that the authors do a great job at situating their work in this literature, by presenting some of the most prominent themes that have emerged so far – this is potentially of great interest to the audience of this journal, that might not be aware of this work.*

*On this basis, the authors have developed a mix of design work and interviews with participants self-tracking for urine analysis. This is very interesting and significant work, and my only suggestion here would be that I would like to know more on the ways in which results from the literature review actually guided the design of the three prototypes.*

**In the section “Indicative user-tests with low fidelity prototypes”, we’ve added a paragraph to better explain how the literature review influenced the design of the prototypes and the user test: *“The challenge of neutrality was a key consideration in the selection of this aspect, as we aimed to examine how our design choices impacted the understandability of the results. The second aspect, meaning-making, was selected based on insights regarding the challenges users face in forming meaningful connections with their data, particularly those related to intention, embodiment, and engagement. Users often struggle to make sense of the data they track and are more likely to engage with their data when it is presented in a way that is meaningful to them. Therefore, we aimed to investigate the quality and potential of the relationship between users and their tracked data by exploring their capacity to relate to different forms of data and form a meaningful connection with it. By adopting an inductive approach we translated these aspects into three parameters that create the differences between the prototypes: levels of abstractions, complexity and numerical precision. Challenges associated with privacy and security were not addressed at this stage of the project due to the low fidelity nature of the prototypes.”***

*The results that the authors collected from the interview and discuss here are also fascinating and very significant for the audience of this journal and beyond. The only suggestion I have on this point concerns the usefulness of raw data and numerical representations. I wonder if the authors can say more on what research participants felt was lacking from raw data and numerical representations and in particular whether they lamented a lack of epistemic tools to understand and process the data as actionable evidence (see e.g. a few papers on this in the context of wearable technologies:* [*https://doi.org/10.1177/2053951720924436*](https://doi.org/10.1177/2053951720924436) *;* [*https://doi.org/10.1371/journal.pdig.0000104*](https://doi.org/10.1371/journal.pdig.0000104)*).*

**We’ve modified a sentence in the section “Preferences and dislikes”: *“In particular, one stated “pH means nothing to me”, expressing a lack of tools for interpretation of the plain results and the need for a more personal explanation of the data and parameters.”***

**We also rewritten the part “Seeing numbers is necessary, but not enough” in the conclusion to better explain the feelings of the participants regarding raw data. We have also included the first of the two references suggested. This part is now written as follow: *“Existing literature has identified challenges that self-tracking users face in making sense of the data they collect (54) and our study further explores these difficulties in the context of health-related data. While our participants appreciated seeing their health data quantified, they also expressed a desire for the information to be presented in a way that was more personally relevant and understandable. Participants noted that medical terminology and raw numerical data can be difficult to process and apply in their daily lives, which may impede their ability to take meaningful action based on the information presented. Therefore, we suggest that for urine home monitoring, data representation should balance numerical representation with qualitative ones, allowing for both precision and personal understandability of the information.”***

## Reviewer #2:

*This paper proposes best practice principles for designing an improved self-tracking user experience (UX) for general mobile health and for urine analysis tracking in particular. They highlight five important different aspects of the self-tracking UX and examine two approaches to measure these aspects. The first is a semi-structured interview to allow enough flexibility (I wished to read this interview but couldn’t find the relevant part in the text. Where is Appendix A?) and let 16 individuals answer it.*

**The script is provided in Appendix A we attach again. We decided to not include transcripts as each interview included very private and personal aspects of the participants lives, as well as many details that could make them recognizable.**

*The second is three different low-fidelity prototypes (two apps and one physical object) to investigate user understanding of their data and engagement level and try them with mock data on 12 individuals. This paper gives a somewhat psychological point of view of what can increase users’ engagement and diligence. I have to say that I’m less proficient with such kinds of papers. My expertise is more computational and thus, my major comment would be regarding the number of interview participants. The authors did not mention how many actual potential target users took part in the interview part (as if 16 is not already extremely low). They only mentioned that they were part of the group (could be only one participant…). It could give some intuition about people’s preferences but drawing conclusions based on such a small dataset is neither sound nor robust IMHO.*

**We’ve added information on the numbers of potential users (10) and project partners (4 actively working on the project, 2 associated). We’ve also added a paragraph to address the small sample:
“*Given that this study was conducted with a limited sample size of only 16 participants, we recognize that the findings may not be representative of the broader population. Nevertheless, the consistency and convergence of responses across participants suggest a significant level of coherence, providing meaningful insights into user attitudes and behaviors towards self-tracking and urine-monitoring technology. These findings have informed our team's subsequent research and design decisions.”***

*I believe the ms. could be improved if more interviewees were recruited for this study. That being said, the title of this ms. suggests that it’s not a quantitative survey but rather qualitative. Since the editor decided to continue with the review process I guess the ms. meets the journal’s quality bar and thus will recommend accepting for publication. Few minor comments the authors may want to address are (I added line number when needed):*

*Figure 1 does not exist.*

**We have updated the order of the figures that was precedently starting from figure 2.**

*Figure 6 is not very informative.*

**We’ve added a self-made illustration (now Fig. 6) next to the original picture to better describe the prototype.**

*References are not consistently formatted.*

**We have reviewed and updated our reference list to be consistent with formatting.**

*As far as I know, unless it’s very seminal, a direct quotation is not a common convention in scientific papers. I would’ve removed it (given the correct reference) or re-write in my own words.*

**We have removed the quote.**

*106 Check references numbering order (14->27..29->19)*

**We have fixed the issue.**

*145 user’s -> users’*

*182 “Privacy and security”regards.. Missing a space*

*484 redundant dot*

*574 mHeatlh typo*

**All these typos have been corrected.**

## Reviewer #3

*Thank you for the opportunity to review “Designing Self-Tracking Experiences: A qualitative study of the perceptions of barriers and facilitators to adopting digital health technology for automatic urine analysis at home.” I enjoyed reading this paper and compliment the authors on drawing together this important work. There is definitely more work to do on understanding the interface between the quantified-self, design, usability, data comprehension, meaning-making, emotional experiences and long-term engagement of users in health devices.*

*The paper is written very well and I have no comments about the writing except I noticed two typos which will be fixed in proofing.*

*Also, I would reference the second sentence in the abstract as it reads like your opinion not objective data.*

**We added a reference to back up our sentence. The sentence is this one: “ *During the COVID-19 pandemic, a wide range of self-monitoring protocols emerged”* and we referenced *1.	Channa A, Popescu N, Skibinska J, Burget R. The Rise of Wearable Devices during the COVID-19 Pandemic: A Systematic Review. Sensors. 2021 Aug 28;21(17):5787.***

*I have a few general concerns about the paper which, in association with the other reviewers and the editor, you may wish to address. I am not sure this journal is actually the best place for this paper. This paper takes a very constructivist approach which may not suit a positivist context. However, I will let the editor make that final decision. None the less this is a high quality paper. Perhaps up front you can signal you are presenting a critical hermeneutic work that is grounded in empirical research. This is an unarticulated strength of this work.*

**We’ve added in the introduction a reference on research through design and an indication regarding the empirical nature of our research.
The new sentence is this: *“In this process, grounded in empirical research, we followed the approach of research through design. This includes investigation to gather diverse perspectives on the problem at hand, reflective practice and the integration of design activities and artifacts as a means of inquiry and knowledge generation.”,* with the following reference**

**9.	Zimmerman J, Forlizzi J, Evenson S. Research through design as a method for interaction design research in HCI. In: Proceedings of the SIGCHI Conference on Human Factors in Computing Systems [Internet]. San Jose California USA: ACM; 2007. p. 493–502. Available from: https://dl.acm.org/doi/10.1145/1240624.1240704**

*Related work*

*Firstly, from my knowledge of the literature in the quantified self space you have done an excellent job at representing the arguments which support and critique self-tracking, particularly in relation to power and control. The thematic synthesis of this review is sound. I personally would prefer to see the section “related work” (e.g. literature review) prior to seeing the context of the study (urine tracking). This would make for a cleaner reading of the review (theory) without going into the context (operationalisation of the theory). Then I would make a clearer argument as to why you chose the context. I believe it is in the paper, just pull it out and make it stronger. This would then provide a link between the literature and context that makes more sense and it wasn’t just a random choice.*

**We’ve addressed this comment in two ways:**

- **In the introduction, we’ve added some information regarding the choice of the context**
- **We’ve moved the introduction part studies on the development of urine tracking devices to the “related work” section. In order to do so, we’ve changed the structure of the sub-headings in this section.**

*Also in the end of the previous work section I would have expected to see the ‘gaps’ in the literature and some research questions you are trying to address.*

**We’ve clarified the gaps at the end of this section: first, the lack of studies on the emotional and the functional performances of different approaches of graphic, interface or information design in the quantified self field. Secondly the lack of studies that have examined the role of design and user experience in the context of urine tracking technologies to evaluate potential barriers or factors affecting acceptance.**

**We also present two research questions:
*- What design approaches influence user experience and adoption of technologies for urine home monitoring?
- What form of data representation is best adapted to the context of urine home monitoring?***

**A clearer reference to these two elements has also been added in the discussion and conclusion.**

*Methods*

*In the methods section I would like to understand more about the thematic analysis. It sounds like you used grounded theory, rather than an a-priori framework, however this should be articulated and codebook made available.* - >

**At the beginning of the semi-structured results, we’ve added a sentence to explain that our thematic analysis indeed followed an inductive approach. We also add the coding table of our interviews in S3 Appendix. We reference the appendix and the choice of the main themes in the section “Thematic analysis from semi-structured interviews”.**

*It is not totally clear how you drew from related work and semi structured interviews (line 276) to develop the parameters of the prototypes. If you can make this clearer in a table which describes comments, codes /themes, design insights and prototype elements that would make for a better link between the literature, interviews and methods.*

**Also following Reviewer #1 comments, as explained before, we’ve added a paragraph to better explain how the literature review influenced the design of the prototypes and the user test.**

*Results*

*It is slightly confusing that the results of the semi-structured interviews are placed after the prototypes are introduced, given they informed the prototypes, however this may only be an issue for me.*

**We’ve decided to leave this part of the paper structure unchanged, as we believe presenting first the methodologies and then all the results allows for an easier and clearer read of the paper.**

*In the results section I might expect to see a return to the literature and address the ‘gaps’ in the literature and an answer to the research questions you proposed based on your literature search. Again I think the information is in the paper, but could be made more explicit.*

**We added a direct reference to the gaps in the discussion, and to our research questions and their answers in the conclusions.**
