# Supplementary material for: Designing self-tracking experiences: A qualitative study of the perceptions of barriers and facilitators to adopting digital health technology for automatic urine analysis at home
Source: PLOS Digit Health. 2023 Sep 15;2(9):e0000319. doi: 10.1371/journal.pdig.0000319 (PMC10503698; doi:10.1371/journal.pdig.0000319)
Supplement: Attachment — Submitted filename: Response to Reviewers.docx [file pdig.0000319.s005.docx]

To whom it may concern,

First of all, we would like to express our gratitude for taking the time to review our work. The editor's insightful feedback and constructive comments have been immensely valuable in shaping and improving our paper.

For clarity, in our response we reported the editors and reviewers’ comments in italics and our answers in bold.

*Additional Editor Comments (if provided):*

*The paper needs a further revision in the Discussion/Conclusion. The discussion must more clearly relate the literature to the current study; i.e. a discussion of this study in relation to what is already known. It may also discuss the limitations of this study.*

*The conclusion should 'wrap up' and close the paper, possibly with key messages and further research. It should not add new findings. Consider moving the content of the conclusion to the discussion.*

**We’ve reworked the discussion and conclusion. We’ve incorporated the findings and answers to the research questions, previously in the conclusion, in the discussion. We removed from the discussion some redundant parts to focus more on our findings and their relationship to what’s already known. We included a paragraph on the intention of our study, including it’s limitations: “As an exploratory study, our focus was on gathering diverse perspectives and generating preliminary insights to inform future investigations. While our insights are limited by the small sample for both the interviews and the user-test, it is important to note that the aim was not to draw definitive conclusions but to uncover emerging patterns and guide further research in the design process. Our study represents an initial step in the research process of designing for automatic urine analysis at home. It therefore serves as a foundation for subsequent iterations that would need to involve larger samples, higher-fidelity prototypes that closely resemble the intended final product, and extended observation periods.”**

**The conclusion was completely rewritten to wrap up the paper.**

*A few minor edits also required across the other sections:*

*Remove the citation from the Abstract [1]
Citations should start from [1] in the introduction*

**We’ve removed the citation in the abstract, so citations start now in the introduction**

line25: traditionalist users - may be taken as judgemental; a more descriptive term could be used instead

**We changed to: *“users more accustomed to traditional, numerical approaches.”***

*55: CSEM - Microtechnolog - a typo? Please consider also providing a URL for more information on this Centre*

**We’ve corrected the typo and added the URL in the manuscript text.**

*69-70: 'we followed the approach of research through design' - needs a reference*

**We’ve added a reference to: Zimmerman J, Forlizzi J. Research Through Design in HCI. In: Olson JS, Kellogg WA, editors. Ways of Knowing in HCI [Internet]. New York, NY: Springer New York; 2014. p. 167–89. Available from: http://link.springer.com/10.1007/978-1-4939-0378-8_8**

*104: 'self-trackers give a lot of value to..' - feels colloquial, consider changing to 'self-trackers value ... '*

**We changed the sentences to: “long-term self-trackers value the support and motivation that these technologies trigger for having a durable change in their lives”**

*221: 'Although there are a lot of interpretations given to such relationship, we can affirm with certainty that the exchange present into this relationship is divisive, as it can be both seen as an empowering energy that enable the user' - the claim for certainty needs to be substantiated (consider rephrasing, and/or add references)*

**We rephrased as follows: “While various interpretations exist regarding this relationship, it can be argued that the exchange within this dynamic is often perceived as divisive, encompassing both empowering and disempowering aspects. On one hand, it can be seen as an empowering energy that enables the user, on the other hand as a limit for the user in their exchange with its body and the world.”**

*421: 'The majority of participants (8/16)' - this appears to be half, not a majority*

**We changed to: “Half of the participants (8/16)”**

*533: 'Many papers note...' - needs references of these papers*

**References have been added to support this sentence with papers previously cited in the literature review.**

561: '...technologies as seen in related work' - needs references to the related work

**We rephrased the sentence to make a cleared connection to the related work section of the paper: “Understanding the complexities and comprehensibility of phenomena can offer potential solutions to the common problems affecting technologies, specifically the ones highlighted in the related work section through the identification of five key challenges: *intention*, *neutrality*, *embodiment*, *engagement*, and *privacy and security*”**
